# Negative Feedback of the cAMP/PKA Pathway Regulates the Effects of Endoplasmic Reticulum Stress-Induced NLRP3 Inflammasome Activation on Type II Alveolar Epithelial Cell Pyroptosis as a Novel Mechanism of BLM-Induced Pulmonary Fibrosis

**DOI:** 10.1155/2022/2291877

**Published:** 2022-08-18

**Authors:** Qiaohui Hong, Yue Zhang, Weixian Lin, Wei Wang, Yafei Yuan, Jiajia Lin, Zhanzhan Xie, Xu Li, Ying Meng

**Affiliations:** ^1^Departments of Respiratory and Critical Care Medicine, Chronic Airways Diseases Laboratory, Nanfang Hospital, Southern Medical University, Guangzhou, China; ^2^Department of Emergency of Medicine, Nanfang Hospital, Southern Medical University, Guangzhou, China

## Abstract

Endoplasmic reticulum stress (ER stress) contributes to the development of pulmonary fibrosis, especially in type II alveolar epithelial cells (AECs) apoptosis. ER stress also promotes NLRP3 inflammasome activation which is inhibited by upregulation of cAMP/PKA pathway. However, it is confused whether ER stress-induced NLRP3 inflammasome activation and pyroptosis in type II alveolar epithelial cells which exacerbates pulmonary fibrosis via a mechanism that is suppressed by cAMP/PKA pathway. In our research, we explored that potential links among NLRP3 inflammasome, ER stress, and cAMP/PKA pathway in type II AECs to explain the new mechanisms of pulmonary fibrosis. We found that in vivo, ER stress, NLRP3 inflammasome, and PKA upregulated in the alveolar epithelial area in animal models of pulmonary fibrosis. In addition, immunofluorescence staining further confirmed that ER stress, NLRP3 inflammasome, and cAMP/PKA had potential links on type II AECs in BLM group. In vitro, ER stress stimulated NLRP3 inflammasome activation, promoted pyroptosis, and also upregulated cAMP/PKA pathway. Upregulation of cAMP/PKA pathway inhibited ER stress-induced pyroptosis of A549 cells and vice versa. These results initially supported conclusion that ER stress may stimulate NLRP3 inflammasome activation and pyroptosis in type II AECs, which exacerbated pulmonary fibrosis, and cAMP/PKA pathway may act as a feedback regulator.

## 1. Introduction

Endoplasmic reticulum stress (ER stress) promotes type II alveolar epithelial cells (AECs) apoptosis, and a mechanism refers to the progression of pulmonary fibrosis [1–3]. However, accumulating evidence showed that ER stress stimulated NLRP3 inflammasome activation which linked to many diseases, such as cardiovascular disease [4], diabetes mellitus [5, 6], and steatohepatitis [7]. Indeed, previous investigations have also confirmed that NLRP3 inflammasome activation may contribute to disease progression in pulmonary fibrosis [8–13]. Therefore, whether ER stress-induced NLRP3 inflammasome activation promotes type II AECs pyroptosis and exacerbates pulmonary fibrosis needs to be established.

Cyclic adenosine monophosphate (cAMP) is a well-characterized second messenger that activates phosphorylation of protein kinase A (PKA) and further regulates cell signaling pathways [14]. In 2012, Lee et al. reported that Ca^2+^ in macrophages inhibited the expression of intracellular cAMP, resulting in NLRP3 inflammasome activation via calcium-sensing receptors [15]. Subsequently, numerous studies have confirmed that cAMP/PKA activation inhibited NLRP3 inflammasome activation [16–20]. Interestingly, ER stress may promote cAMP/PKA activation [21, 22]. Thus, we speculated that the negative feedback of the cAMP/PKA pathway regulated ER stress-induced NLRP3 inflammasome activation and pyroptosis in type II AECs which may act as a novel mechanism of pulmonary fibrosis.

In this study, we explored whether ER stress-induced NLRP3 inflammasome activation promotes type II AECs pyroptosis in a mouse model of bleomycin- (BLM-) induced pulmonary fibrosis. Furthermore, we also investigated whether ER stress upregulated cAMP/PKA pathway and whether upregulation of the cAMP/PKA pathway suppressed ER stress-induced NLRP3 inflammasome activation and pyroptosis in type II AECs which alleviated pulmonary fibrosis.

## 2. Methods

### 2.1. Ethics Statement

Animal experiments were approved by ethics committee of Nanfang hospital and conform to the relevant norms of animal ethics.

### 2.2. Animal Experiments

Male C57/BL mice (aged 5–8 weeks) were obtained from animal breeding facility of Southern Medical University. Mice were divided into two groups with 10 mice per group: the BLM group and the control group. Mice in the BLM group were given 5 mg/kg bleomycin through tracheal administration under anesthesia; the control group was given the equivalent amount sterile saline in the same way. All mice were sacrificed after 28 days.

### 2.3. Histological and Immunohistochemical Analyses

Immunohistochemical staining of paraffin-embedded lung sections (4 *μ*m) was performed with primary antibodies against Grp78, Grp94, CHOP, NLRP3, IL-1*β*, ASC, GSDMD, and PKA (1: 200, Proteintech, China). Immunoreactivity was visualized using a commercial HRP-based method (GTVision TM Detection System/Mo&Rb, Denmark).

### 2.4. Immunofluorescence Histochemistry

Immunofluorescent staining of lung sections (4 *μ*m) was performed by incubation overnight in low temperature with combinations of detective primary antibodies. After being hatched with different species and different wavelengths of secondary antibodies, images were visualized under fluorescence microscope.

### 2.5. Immunocytochemistry

Cells cultured on glass coverslips were fixed with paraformaldehyde, ruptured membrane with Triton X-100 and sealed with FBS (fetal bovine serum), and then hatched overnight at 4°C in a moist chamber with the primary antibodies. PBST was used to clean the primary antibody and hatch the fluorescent secondary antibody. Finally, images were observed by fluorescence microscope within 48 hours.

### 2.6. LDH Release Assay

LDH was released following cell death because of disruption of the plasma damage. Cell viability was assessed by the concentration of LDH release which was detected by LDH cytotoxicity assay detection kit (Beyotime, China).

### 2.7. CCK8 Assay

Cell viability was evaluated by CCK8 assay (FUDE, China).

### 2.8. cAMP Measurement

The concentration of cAMP was determined using an ELISA kit (SAB, America).

### 2.9. siRNA-Mediated Silencing of NLRP3

A549 cells were incubated into 6- or 96-well plates and transfected with a NLRP3 siRNA (siNLRP3 sense: 5′CCAAGAAUCCAAGUGUAATT 3′; siNLRP3 antisense: UUACACUGUGGAUUCUUGGCT) or a negative control using Lipofectamine 3000 (Invitrogen, America). Transfection efficiency was evaluated by Western blot and qPCR analyses.

### 2.10. Western Blot Analysis

Western blot was performed in a standardized procedure. PVDF membranes were incubated with specific antibodies against Grp94, CHOP, NLRP3, IL-1*β*, caspase-1, GSDMD, PKA, cleaved IL1*β* (1 : 1,000, Proteintech, China), cleaved IL1*β* (1 : 10,00, Bioss, China), and GADPH (1 : 10,000, FUDE, China).

### 2.11. Real-Time Quantitative-PCR Analysis

Total RNA was extracted in a standardized procedure with the following normalized detection methods. The expression of mRNA was normalized against GADPH.

### 2.12. Statistical Analysis

*T*-test was utilized to evaluate differences between groups and ANVOA to evaluate differences between more than two groups. *P* < 0.05 was considered as significant difference.

## 3. Results

### 3.1. Elevation of ER Stress and NLRP3 Inflammasome in the Mouse Model of BLM-Induced Pulmonary Fibrosis

**Figure 1:**
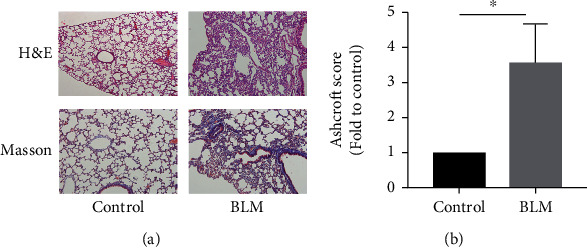
(a) H&E and Masson's trichrome staining (magnification 200×). (b) Relative Ashcroft score. ∗*P* < 0.05 vs. control group.

**Figure 2:**
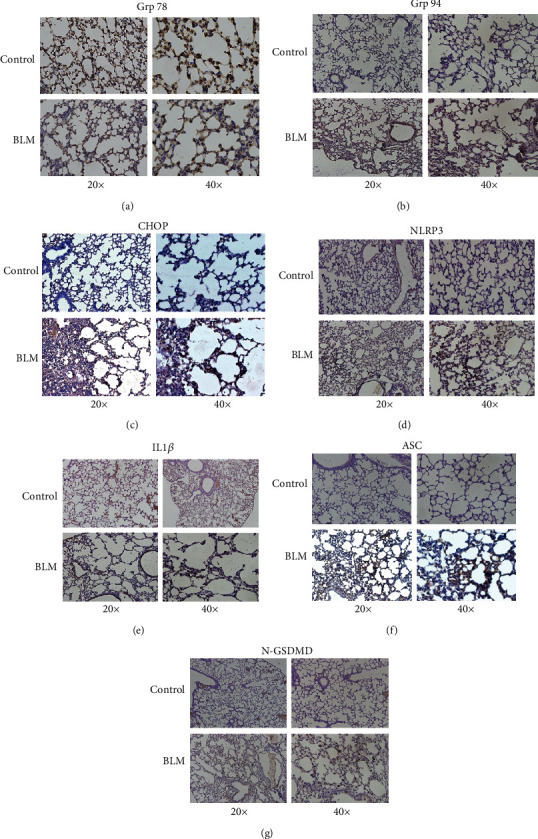
ER stress and NLRP3 inflammasome activation increased in the alveolar region in the BLM group. (a–c) Immunochemical staining of ER stress markers Grp78, Grp94, and CHOP and (d–g) NLRP3 inflammasome-related proteins NLRP3, IL1*β*, ASC, and N-GSMDM.

BLM-induced pulmonary fibrosis in mice was detected by hematoxylin and eosin and Masson's trichrome staining (Figure 1(a)). The fibrosis score was also increased in BLM-induced pulmonary in mice compared with control group (Figure 1(b)). Immunochemical staining (Figures 2(a)–2(c)) of ER stress markers in the alveolar region revealed decreased expression of Grp78 in the BLM model group, while expression of Grp94 and CHOP increased, suggesting that loss of the chaperone protein Grp78 promoted ER stress, which was consistent with previous reports [23]. Simultaneously, compared with the control group, elevated alveolar expression of the NLRP3 inflammasome-related protein NLRP3, IL-1*β*, ASC, and N-GSDMD was detected in the BLM model group (Figures 2(d)–2(g)). Collectively, these observations indicated elevations of ER stress and NLRP3 inflammasome activation in BLM-induced pulmonary fibrosis.

### 3.2. Coexpression of ER Stress Marker and NLRP3 Inflammasome-Related Proteins in Type II Alveolar Epithelial Cells in BLM-Induced Pulmonary Fibrosis

**Figure 3:**
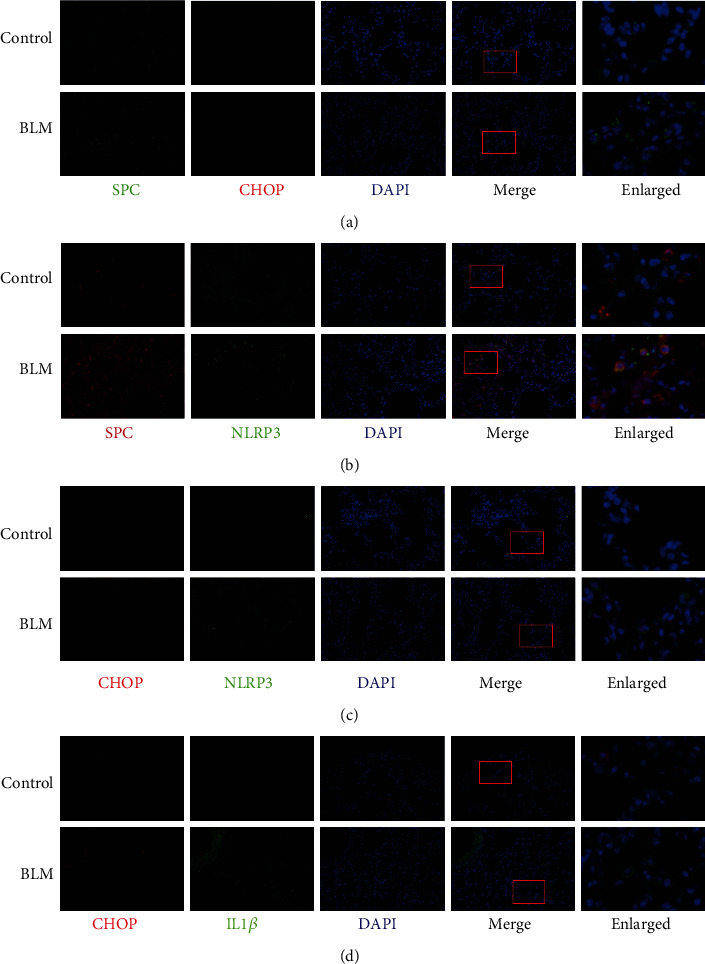
Coexpression of ER stress marker and NLRP3 inflammasome-related proteins in type II alveolar epithelial cells in BLM-induced pulmonary fibrosis. Immunofluorescence staining showing (a, b) colocalization of SPC with the ER stress marker CHOP and the NLRP3 inflammasome-related protein NLRP3 and (c, d) colocalization of the ER stress marker CHOP with the NLRP3 inflammasome-related protein NLRP3 and IL-1*β*.

Double-staining showed that the expression of the ER stress marker CHOP and NLRP3 colocalized with surfactant protein C (SPC) in the BLM model group, indicating that ER stress and NLRP3 inflammasome activation were closely linked in type II AECs in BLM-induced pulmonary fibrosis (Figures 3(a) and 3(b)). This conclusion was further supported by the increased colocalization of CHOP and NLRP3 with IL-1*β* (Figures 3(c) and 3(d)).

### 3.3. Endoplasmic Reticulum Stress Promoted NLRP3 Inflammasome Activation and Pyroptosis in Type II Alveolar Epithelial Cells

**Figure 4:**
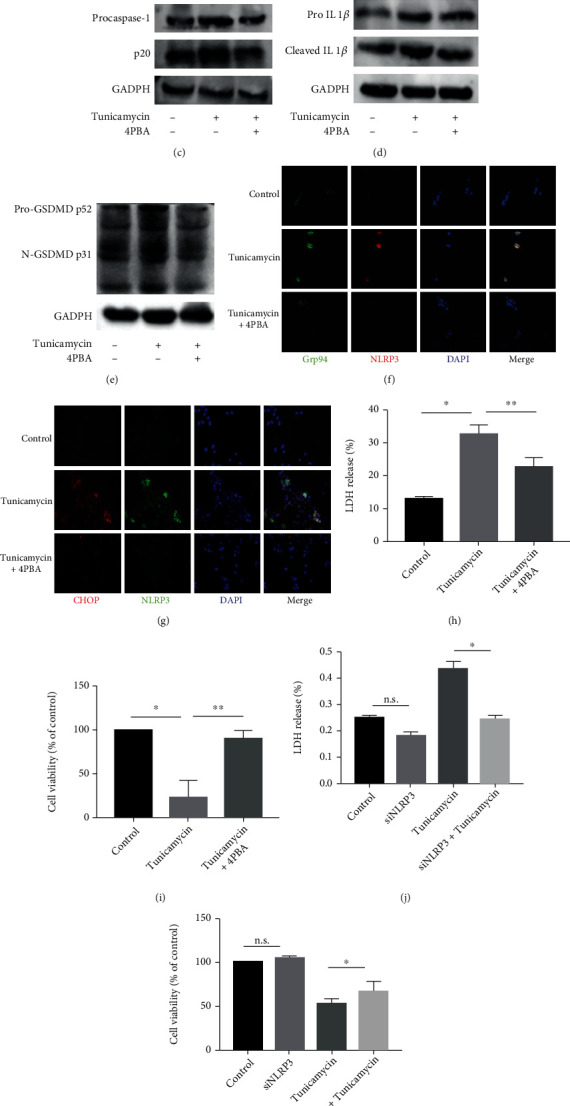
Endoplasmic reticulum stress promoted NLRP3 inflammasome activation and pyroptosis in type II alveolar epithelial cells. A549 cell lines were pretreated 4PBA (5 mM) for 1 h or NLRP3 expression was knocked down by siRNA transfection before exposure to tunicamycin (1 *μ*g/mL) for 24 h. (a–e) Protein levels of the ER stress markers Grp94 and CHOP and the NLRP3 inflammasome-related proteins NLRP3, ASC, cleaved caspase-1, cleaved IL1*β*, and N-GSDMD were detected by Western blot analysis. (f, g) Colocalization of Grp94/NLRP3 and CHOP/NLRP3 was detected by immunofluorescence staining. (h) LDH release assay of cell viability. (i) Cell viability was assessed by CCK8 assay. ^∗^*P* < 0.05 vs. control group, ^∗∗^*P* < 0.05 vs. tunicamycin group, (j) LDH release assay of cell viability, (k) cell viability was assessed by CCK8 assay. n.s.: not significant vs. control group, ^∗^*P* < 0.05 vs. tunicamycin group.

To investigate whether ER stress induced-NLRP3 inflammasome activation in type II AECs, we stimulated A549 cells with tunicamycin (the ER stress promoter) in vitro. Western blot analysis showed that tunicamycin increased the expression of the ER stress marker proteins Grp94 and CHOP, and the NLRP3 inflammasome-associated proteins NLRP3, ASC, cleaved caspase-1, cleaved IL-1*β*, and N-GSDMD compared with the control group, while these changes were suppressed by 4PBA (ER stress inhibitor) (Figures 4(a)–4(e), Supplementary Figure 1). After stimulation of A549 cells with tunicamycin for 24 h, RT-qPCR analysis showed that the relative mRNA expression levels of Grp94, CHOP, NLRP3, ASC, IL1*β*, and IL18 were increased compared with control group, and these changes were suppressed by 4PBA (Supplementary Figure 2). In addition, double immunofluorescence staining showed that the ER stress markers Grp94 and CHOP colocalized with NLRP3 in A549 cells simulated with tunicamycin, which further confirmed ER stress induced-NLRP3 inflammasome activation (Figures 4(f) and 4(g)). Furthermore, LDH release and CCK8 assays revealed that LDH release was increased while cell viability was decreased stimulated with tunicamycin compared with the control groups (Figures 4(h) and 4(i)). Indeed, after confirming siRNA-mediated knockdown of NLRP3 expression in A549 cells by Western blot and RT-qPCR analyses (Supplementary Figure 3), we showed that NLRP3 knockdown inhibited the release of LDH and decrease of cell viability stimulated by tunicamycin, indicating that ER stress promoted cell pyroptosis (Figures 4(j) and 4(k)).

#### 3.3.1. Activation of the cAMP/PKA Pathway in BLM-Induced Pulmonary Fibrosis

**Figure 5:**
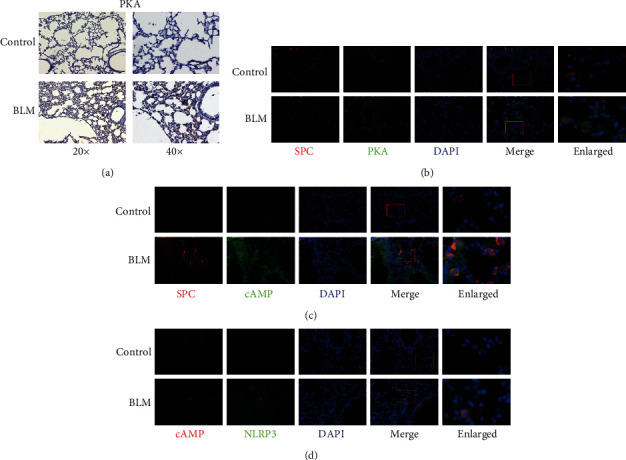
Upregulation of the cAMP/PKA pathway in BLM-induced pulmonary fibrosis. (a) Immunohistochemical staining of PKA protein. Immunofluorescence staining showing colocalization of (b, c) SPC/PKA and SPC/cAMP and (d) cAMP/NLRP3.

Immunochemical staining revealed the expression of PKA elevated in the BLM model group compared with the control group (Figure 5(a)). Double immunofluorescence staining showed that colocalization of SPC with cAMP and PKA increased in the BLM model group (Figures 5(b) and 5(c)). Interestingly, colocalization of NLRP3 with cAMP also increased in the BLM model group, indicating a potential link between the cAMP/PKA pathway and the NLRP3 inflammasome in type II AECs in BLM-induced pulmonary fibrosis.

### 3.4. ER Stress Induced Upregulation of the cAMP/PKA Pathway in Type II AECs

**Figure 6:**
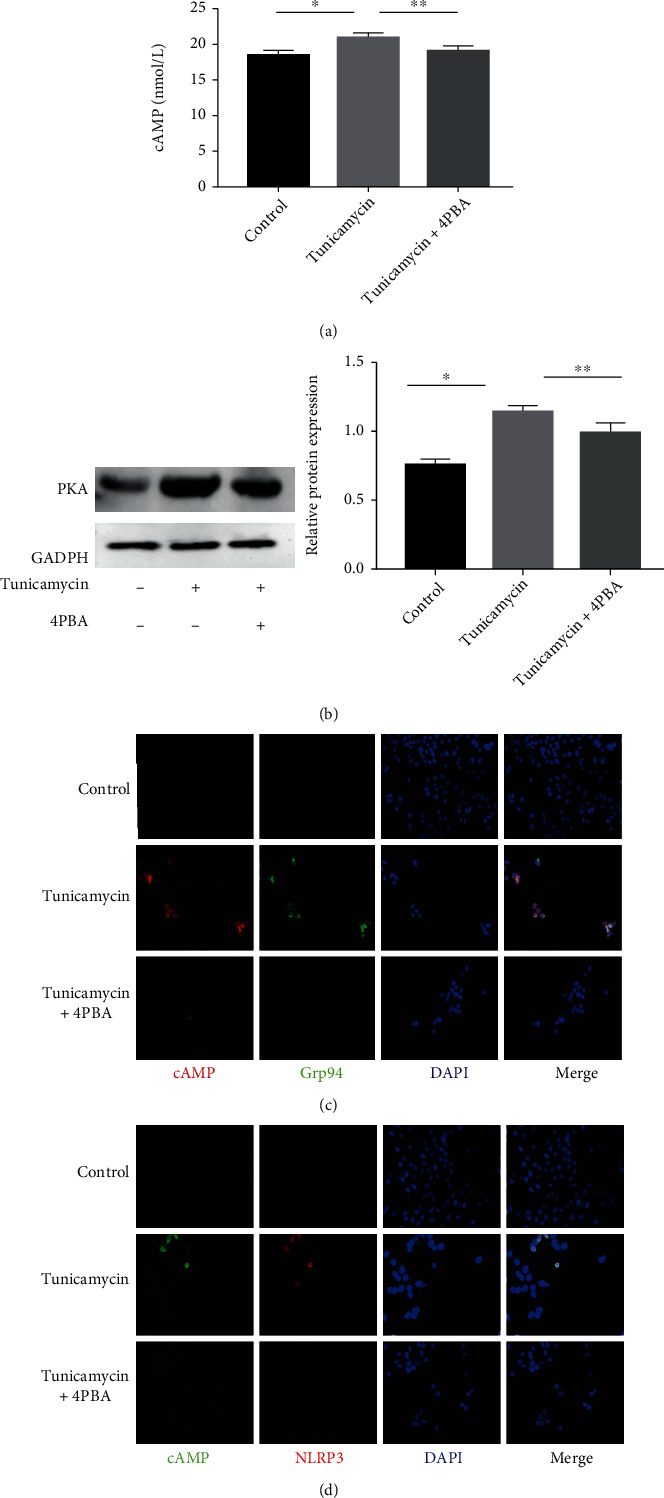
ER stress induced upregulation of the cAMP/PKA pathway. (a) A549 cells were pretreated with 4PBA (5 mM) for 1 h before exposure to tunicamycin (1 *μ*g/mL) for 24 h. cAMP was detected by ELISA. (b) Western blot showed PKA protein level. (c, d) Immunofluorescence staining showed colocalization of cAMP/Grp94 and cAMP/NLRP3. ^∗^*P* < 0.05 vs. control group, ^∗∗^*P* < 0.05 vs. tunicamycin group.

Compared with the control group, intracellular cAMP levels and the expression of PKA protein were increased in the tunicamycin group (Figures 6(a) and 6(b)). Double immunofluorescence staining showed that the ER stress marker Grp94 colocalized with cAMP and NLRP3 colocalized with cAMP (Figures 6(c) and 6(d)). Collectively, these results indicated that ER stress induced activation of the cAMP/PKA pathway and that the cAMP/PKA pathway was closely associated with the NLRP3 inflammasome.

### 3.5. Inhibition of the cAMP/PKA Pathway Exacerbated Type II AEC Death Induced by ER Stress

**Figure 7:**
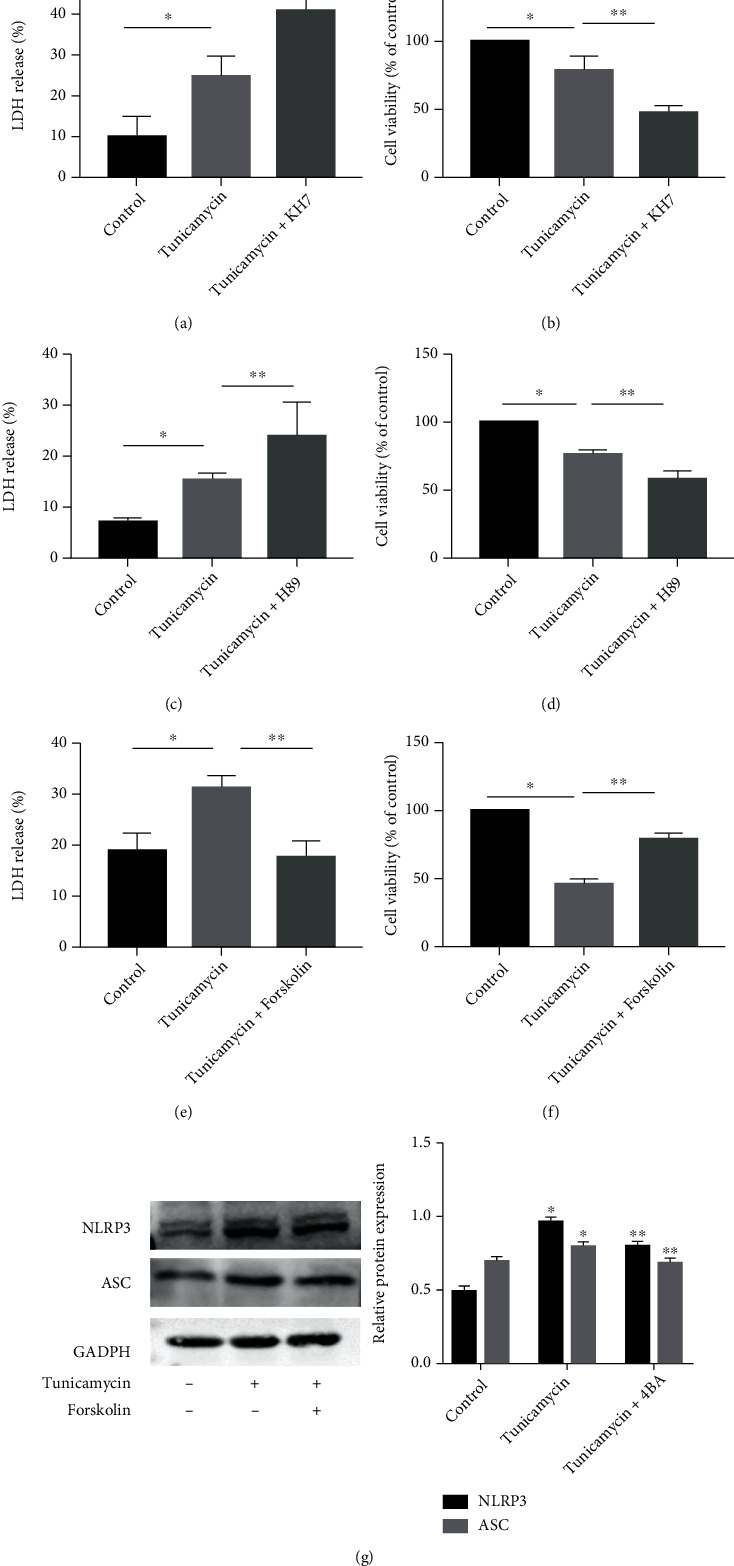
Inhibition of the cAMP/PKA pathway exacerbated type II alveolar epithelial cell death induced by ER stress. A549 cells were pretreated KH7 (10 *μ*M) or H89 (8 *μ*M) or forskolin (100 *μ*M) for 1 h before exposure to tunicamycin for 24 h. (a) LDH release assay of cell viability. (b) Cell viability was assessed by CCK8 assay. (c) LDH release assay of cell viability. (d) Cell viability was assessed by CCK8 assay. (e) LDH release assay of cell viability. (f) Cell viability was assessed by CCK8 assay. ^∗^(g) Western blot showing NLRP3 and ASC protein levels; ^∗^*P* < 0.05 vs. control group, ^∗∗^*P* < 0.05 vs. tunicamycin group.

We pretreated A549 cells with KH7 (adenylate cyclase inhibitor) and H89 (PKA inhibitor) before tunicamycin treatment. KH7 and H89 further increased the LDH release and decreased the cell viability induced by ER stress (Figures 7(a)–7(d)). In addition, we found that the adenylate activator forskolin reversed the release of LDH and decrease of cell viability induced by ER stress (Figures 7(e) and 7(f)). Forskolin attenuated the levels of NLRP3 and ASC induced by ER stress (Figure 7(g)). These results indicated that the cAMP/PKA pathway regulated ER stress-induced type II AEC pyroptosis via a negative feedback mechanism.

## 4. Discussion

**Figure 8:**
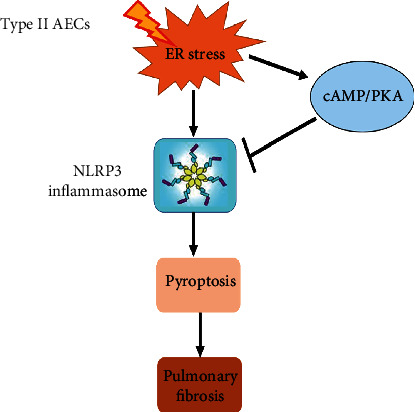
Negative feedback of the cAMP/PKA pathway regulates the effects of ER stress-induced NLRP3 inflammasome activation on type II alveolar epithelial cell pyroptosis, which alleviates pulmonary fibrosis.

Our current study confirmed the potential links among ER stress, NLRP3 inflammasome, and cAMP/PKA pathway in type II AECs in pulmonary fibrosis. We further demonstrated that ER stress induced-NLRP3 inflammasome activation upregulated cAMP/PKA pathway and pyroptosis in A549 cells. In addition, we found that inhibition of the cAMP/PKA pathway further promoted ER stress induced-pyroptosis. These findings indicated that ER stress promoted NLRP3 inflammasome activation and further facilitated pyroptosis in type II AECs which resulted to pulmonary fibrosis progression, and cAMP/PKA may act as a negative feedback regulator (Figure 8).

ER stress-induced apoptosis of type II AECs has been considered to be a crucial component of the mechanism of pulmonary fibrosis [1–3]. However, numerous research have found that ER stress could facilitate NLRP3 inflammasome activation by promoting upregulation of TXNIP [5, 6], accumulation of ROS [24], activation of NF-*κ*B [25], potassium efflux [26], and calcium influx [27]. Thus, we suspected that there might be a similar mechanism in pulmonary fibrosis, such as ER stress facilitating AECs pyroptosis by promoting NLRP3 inflammasome. Compared with apoptosis, an immune-silent programmed death [28], pyroptosis could release IL1*β* and IL18 [29] which could interact with macrophages and fibroblast and therefore have more a strong effects on mechanism of pulmonary fibrosis.

The adenylate cyclase activator forskolin has been detected to alleviate the progression of pulmonary fibrosis [30]. Importantly, it has been reported that cAMP levels was decreased in lung fibroblasts in IPF due to repression of G*α*_s_-coupled receptors [31–33]. Furthermore, many studies found that stimulation of the cAMP/PKA pathway suppressed NLRP3 inflammasome activation [15–20]. Interestingly, ER stress may promote cAMP/PKA pathway activation [21, 22]. Simultaneously, we also found that ER stress promoted cAMP/PKA pathway activation in type II AECs. Furthermore, cAMP/PKA pathway activation inhibited ER stress-induced NLRP3 inflammasome activation and cell pyroptosis, while inhibition of the cAMP/PKA pathway further exacerbated ER stress-induced cell death. Our observations indicated that upregulation of cAMP/PKA pathway may alleviate pulmonary fibrosis through inhibiting ER stress-induced NLRP3 inflammasome activation in type II AECs.

Our study has certain deficiencies, which needs to be further explored. A549 cell line is a neoplastic cell line, although several researches have used it as a type II AECs [34, 35]. In addition, it is unclear whether the upregulation of cAMP/PKA pathway could suppress BLM-induced pulmonary fibrosis through inhibiting ER stress-induced pyroptosis of type II AECs. Furthermore, it is unknown the mechanism by which ER stress upregulates cAMP/PKA pathway.

In summary, our study initially confirmed ER stress-induced NLRP3 inflammasome activation in type II AECs, promoted cell pyroptosis, and ultimately exacerbated pulmonary fibrosis. Furthermore, we also indicated that cAMP/PKA may act as a negative feedback regulator of ER stress-induced NLRP3 inflammasome activation, thereby inhibiting type II AEC pyroptosis and eventually alleviating pulmonary fibrosis. These findings gave new insights into the mechanism of pulmonary fibrosis.

## Data Availability

The data used to support the findings of this study are included within the article.

## Supplementary Materials

Supplementary Materials Supplementary Figure 1: changes of NLRP3-related protein levels in type II alveolar epithelial cells induced by ER stress. (a–e) Relative protein levels were detected by Western blot. ^∗^*P* < 0.05 vs. control group; ^∗∗^*P* < 0.05 vs. tunicamycin group. Supplementary Figure 2: changes of NLRP3-related mRNA levels in type II alveolar epithelial cells induced by ER stress. (a–f) Relative mRNA levels were detected by RT-qPCR. ^∗^*P* < 0.05 vs. control group; ∗∗*P* < 0.05 vs. tunicamycin group. Supplementary Figure 3: changes of NLRP3 protein and mRNA levels after NLRP3 knockdown. (a) Western blot showing NLRP3 protein level. ^∗^*P* < 0.05 vs. control group; ^∗∗^*P* < 0.05 vs. tunicamycin group. (b) Relative expression of NLRP3 mRNA was detected by RT-qPCR. ^∗^*P* < 0.05 vs. control group ^∗∗^*P* < 0.05 vs. tunicamycin group.
