# Supplementary material for: Negative Feedback of the cAMP/PKA Pathway Regulates the Effects of Endoplasmic Reticulum Stress-Induced NLRP3 Inflammasome Activation on Type II Alveolar Epithelial Cell Pyroptosis as a Novel Mechanism of BLM-Induced Pulmonary Fibrosis
Source: J Immunol Res. 2022 Aug 18;2022:2291877. doi: 10.1155/2022/2291877 (PMC9410862; doi:10.1155/2022/2291877)
Supplement: Supplementary Materials — Supplementary Figure 1: changes of NLRP3-related protein levels in type II alveolar epithelial cells induced by ER stress. (a–e) Relative protein levels were detected by Western blot. ∗P < 0.05 vs. control group; ∗∗P < 0.05 vs. tunicamycin group. Supplementary Figure 2: changes of NLRP3-related mRNA levels in type II alveolar epithelial cells induced by ER stress. (a–f) Relative mRNA levels were detected by RT-qPCR. ∗P < 0.05 vs. control group; ∗∗P < 0.05 vs. tunicamycin group. Supplementary Figure 3: changes of NLRP3 protein and mRNA levels after NLRP3 knockdown. (a) Western blot showing NLRP3 protein level. ∗P < 0.05 vs. control group; ∗∗P < 0.05 vs. tunicamycin group. (b) Relative expression of NLRP3 mRNA was detected by RT-qPCR. ∗P < 0.05 vs. control group ∗∗P < 0.05 vs. tunicamycin group. [file 2291877.f1.docx]

**Supplementary data**


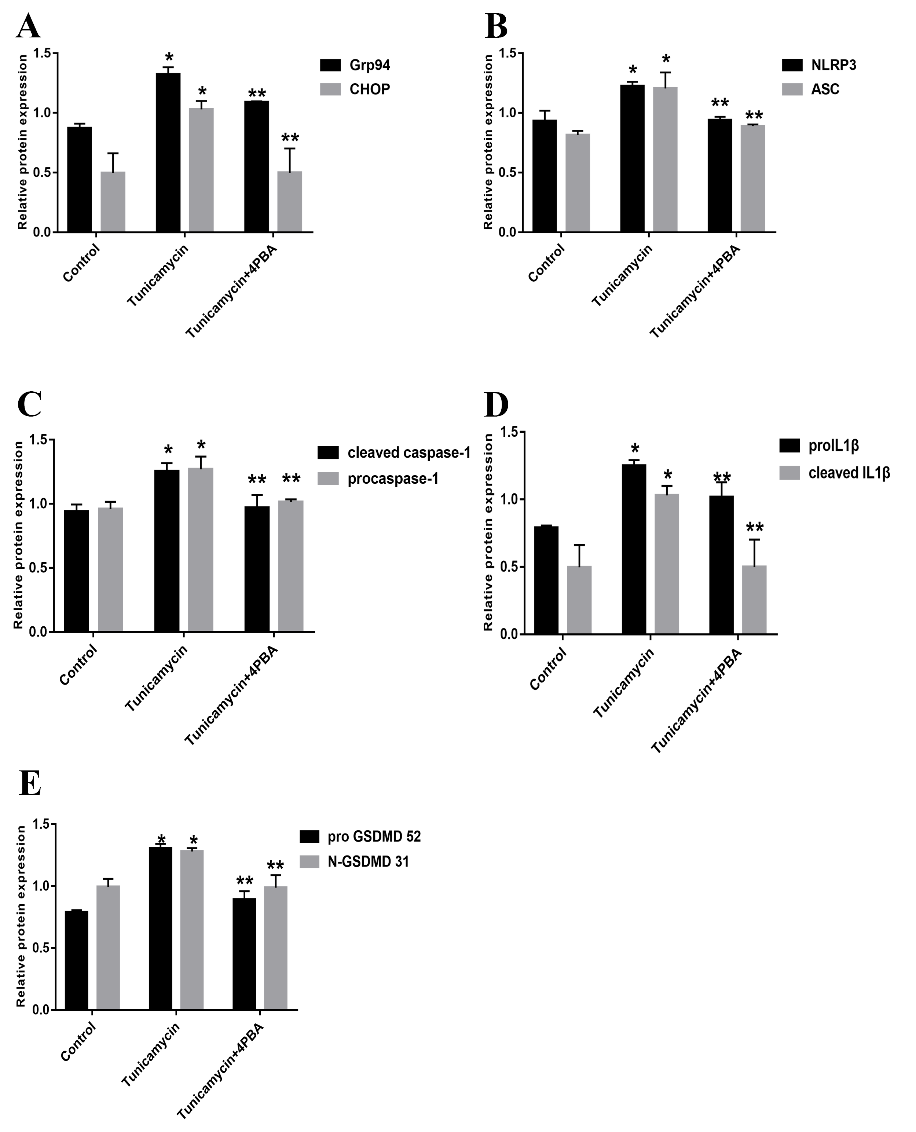


Supplementary Figure 1 (A–E) Relative protein levels were detected by Western blot. ******P*＜0.05 *vs*. Control group ** *P*＜0.05 *vs.* Tunicamycin group


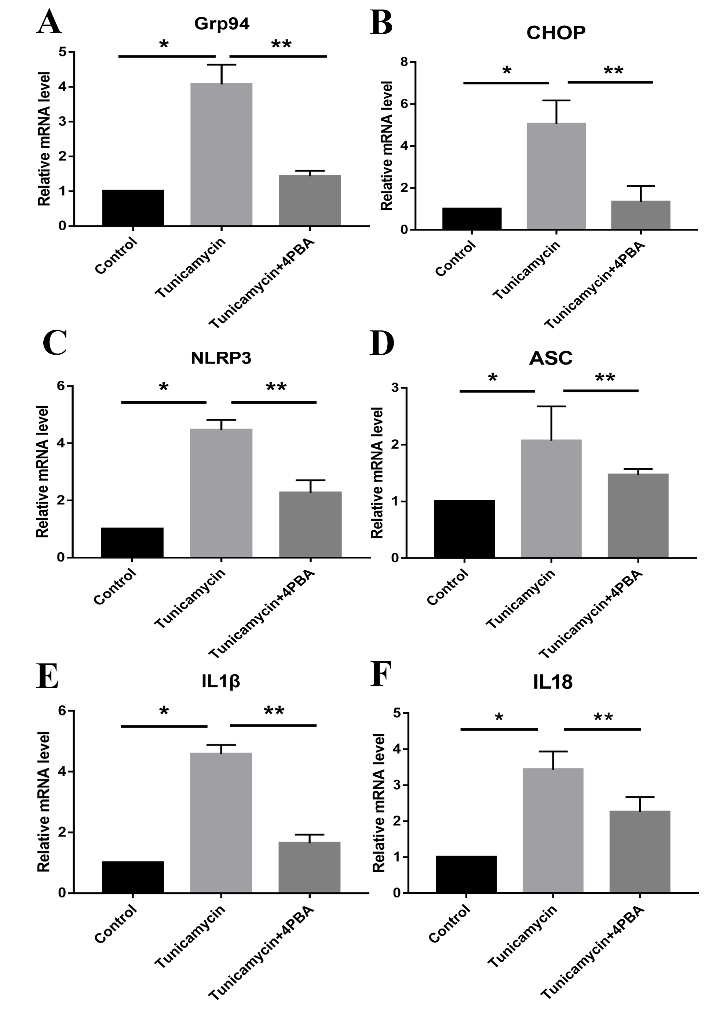


Supplementary Figure 2 (A–F) Relative mRNA levels were detected by RT-qPCR. ******P*＜0.05 *vs*. Control group; ** *P*＜0.05 *vs.* Tunicamycin group


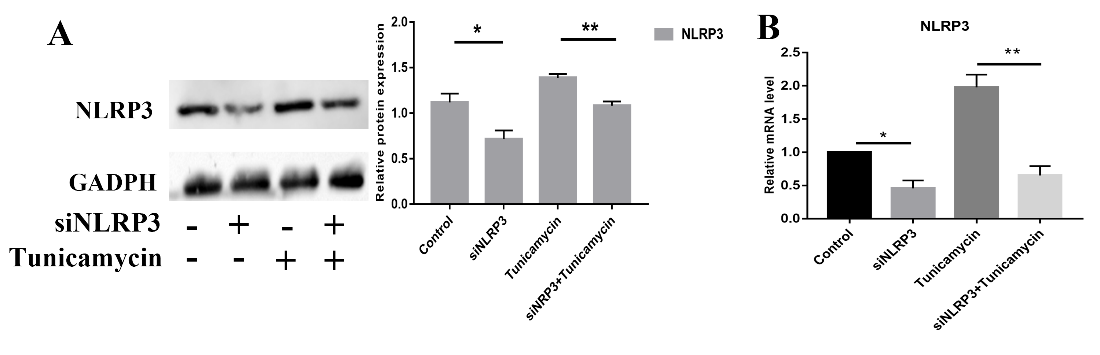


Supplementary Figure 3 (A) Western blot showing PKA protein level ******P*＜0.05 *vs*. Control group ** *P*＜0.05 *vs.* Tunicamycin group (B) Relative expression of NLRP3 mRNA was detected by RT-qPCR. ******P*＜0.05 *vs*. Control group ** *P*＜0.05 *vs.* Tunicamycin group
